# Klotho KL-VS haplotype does not improve cognition in a population-based sample of adults age 55–87 years

**DOI:** 10.1038/s41598-021-93211-x

**Published:** 2021-07-05

**Authors:** Bernhard W. Müller, Anke Hinney, Norbert Scherbaum, Christian Weimar, Christoph Kleinschnitz, Triinu Peters, Lara Hochfeld, Sonali Pechlivanis, Andreas Stang, Martha Jokisch, Bernd Kowall

**Affiliations:** 1 Department for Psychiatry and Psychotherapy, LVR-Hospital, University of Duisburg-Essen, University Hospital Essen, Essen, Germany; 2 Department of Psychology, University of Wuppertal, Wuppertal, Germany; 3 Department of Child and Adolescent Psychiatry, Psychosomatics and Psychotherapy, University of Duisburg-Essen, University Hospital Essen, Essen, Germany; 4 Department of Addictive Behavior and Addiction Medicine, LVR-Hospital, University of Duisburg-Essen, University Hospital Essen, Essen, Germany; 5 BDH-Klinik Elzach gGmbH, Elzach, Germany; 6 Institute for Medical Informatics, Biometry and Epidemiology, University Hospital of Essen, Essen, Germany; 7 Department of Neurology, University Hospital Essen, University of Duisburg-Essen, Essen, Germany; 8 Institute of Human Genetics, University Hospital of Bonn, Bonn, Germany; 9 Institute for Asthma and Allergy Prevention, Helmholtz Zentrum München, German Research Centre for Environmental Health, Munich, Germany

**Keywords:** Psychology, Geriatrics, Public health, Haplotypes

## Abstract

The heterozygous human Klotho KL-VS haplotype has been associated with improved cognitive performance but results are inconsistent. Here we assessed Klotho KL-VS haplotype and cognition using data from the third examination of the population-based Heinz Nixdorf Recall Study. We analyzed cognition tests (immediate and delayed word list, Trail-Making Test [TMT] part A and B, Maze test, interference condition of the Stroop color-word test, verbal fluency) and their associations with Klotho KL-VS haplotype. The Klotho KL-VS haplotype is classified by the V-allele at SNP rs9536314 (F352V) and the S-allele at SNP rs9527025 (C370S). Heterozygotes for the KL-VS haplotype were compared with non-carriers. Analyses were performed in 1812 subjects (55–87 years). We found consistent but only slightly lower performance in heterozygous carriers of the KL-VS haplotype in all tasks with Z-scores ranging between Z = − 0.042 (verbal fluency) and − 0.17 (TMT part A). Differences between carriers and non-carriers were similar for men and women for all tests but TMT part B (interaction contrast = 8.4 s (95% CI − 2.3; 19.1)). While cognition declined with age, we found an effect modification by age (55–65 years, 66–75 years, > 75 years). In the 66–75 years KL-VS heterozygous age group, lower performance was seen in memory, visual attention and motor speed. Contrary to our hypothesis, heterozygous carriers of the KL-VS haplotype did not show enhanced performance in cognitive tests in our study.

## Introduction

Klotho is a protein related to slower aging in animals^1^ and humans^2^. Subforms of Klotho have been identified. Alpha-Klotho is the most prominent isoform and will here be referred to as Klotho^3^. Klotho interacts with insulin and Wnt signaling pathways, inhibits oxidative stress and regulates phosphatase and calcium absorption^4^. Klotho is circulating in blood, but has also been identified in cerebrospinal fluid (CSF)^5^. A possible mechanism of the central action of Klotho was implied by a mouse model for Alzheimer’s disease. Increased mouse CSF Klotho levels prevented depletion of a NMDA receptor subunit in the hippocampus and enhanced spatial learning and memory^6^.

The human Klotho gene (*KL*) is encoded on chromosome 13q12^2,7^. A minor KL variant haplotype KL-VS is defined by six sequence variants (single nucleotide polymorphisms, SNPs) in perfect linkage disequilibrium (LD) with each other. Two of these missense variants lead to the substitution of amino acids (c.1062T->G, F352V, rs9536314; c.1109G->C, C370S, rs9527025) and therefore possibly to effects on protein function. Consistent with a functional effect, increased serum Klotho levels were detected in heterozygous VS carriers^8^. Subjects heterozygous for the KL-VS haplotype (about 25% of Caucasians) showed an increased life span^2^ and a decreased risk for cardiovascular disease^9^. Homozygotes for KL-VS are found in approximately 1.1% of Caucasians and life span was decreased in homozygous KL-VS carriers^2^.

In a longitudinal community study of about 800 adults aged 65 and above, the InCHIANTI study reported that lowered circulating blood Klotho was associated with increased mortality^10^ and lowered grip strength^11^. Moreover, in this sample increased blood Klotho concentrations were associated with a slower decline in Mini-Mental-Status Examination scores, a screening instrument, used to assess gross cognition impairments related to dementia^12^.

With regard to the Klotho KL-VS gene, Dubal et al.^8^ for the first time reported better cognition in heterozygous KL-VS carriers in three independent elderly samples comprising 718 participants. In a more recent study, Morar et al.^13^ reported better memory performance in a sample of 316 younger, but not in 600 elderly healthy heterozygous KL-VS adults. Using the Mini-Mental-Status Examination and additional cognition tests in 1480 very old subjects, Mengel-From et al.^14^ found no beneficial effect of the heterozygous KL-VS haplotype in a large Danish cohort study. A recent study comprising 1387 children and adolescents reported better executive functioning, attention, episodic memory and general cognition only in children below the age of 11 years, a result which however was not replicated in an additional sample of about 2300 children between the age of 6–12 years^15^. Given inconclusive results in these studies with varying sample sizes and different cognition measures, here we aimed to further assess the effect of Klotho KS-VS haplotype on cognitive function in a larger cohort of older subjects, using data from a population-based study in Germany.

## Materials and methods

### Study population

**Figure 1:**
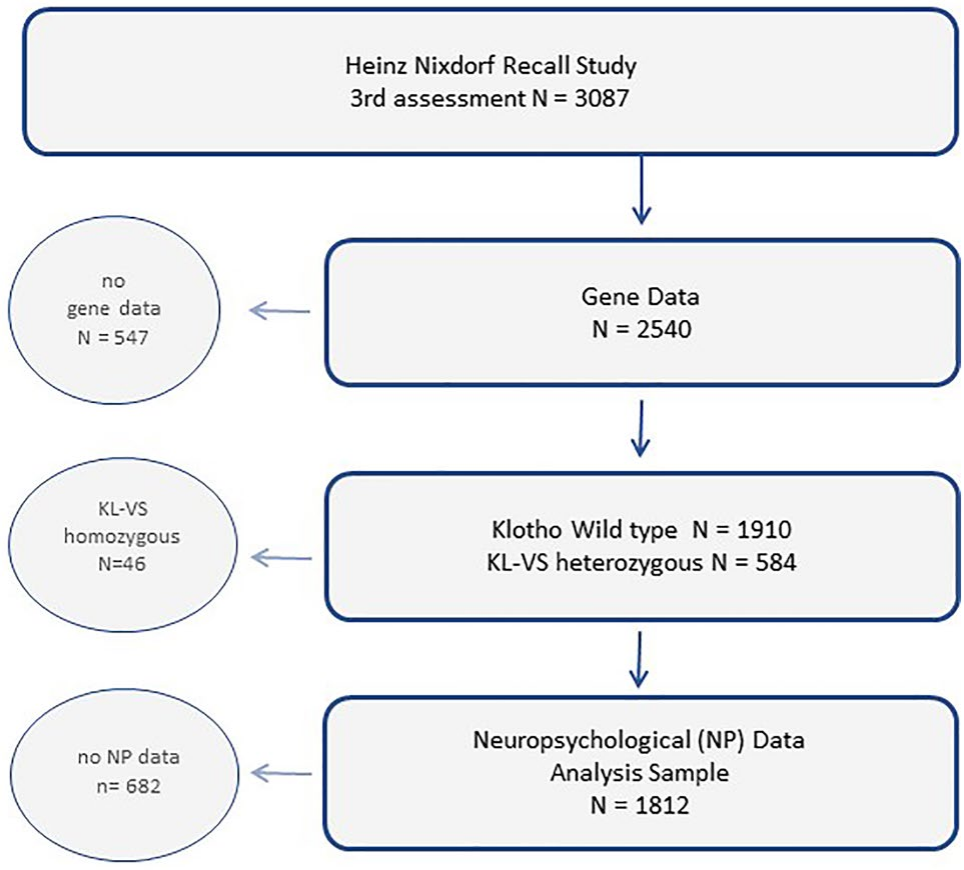
Subject flow chart. The numbers regarding the neuropsychological assessment (NP) give the maximum N. Additional drop outs apply with individual tests. Details on these are given in the “Methods” section.

The Heinz Nixdorf Recall Study is a population-based prospective cohort study conducted in three large adjacent cities (Bochum, Essen, Mülheim) in the Ruhr area in North-Rhine-Westphalia in Germany focusing on heart disease. The study rationale and design have been described in detail elsewhere^16^. In short, the cohort comprises a total of 4814 subjects (49.8% men, aged 45–75 years). The baseline visits were performed between 2000 and 2003. The follow-up visits took place between 2005 and 2008, and the second follow-up visits took place between 2011 and 2015. The median follow-up was 5.1 years for the first follow-up period, and 5.2 years for the second follow-up period. Data assessment at baseline and at follow-up visits included self-administered questionnaires, face-to-face interviews, and a physical examination including among others anthropometric measurements and comprehensive laboratory tests. The third examination in the Heinz Nixdorf Recall Study included 3087 subjects of whom 1812 subjects formed the analysis set (flow chart in Fig. 1). Subjects were split in three groups 55–65 years, 66–75 years and 76–87 years. This allows for the assessment of age as an effect modifier of Klotho—cognition associations and to reveal probable linear, as well as non-linear interaction effects. The study was approved by the ethics committee of the Medical Faculty of the University of Duisburg-Essen. All assessments and methods were carried out in accordance with approved guidelines and regulations and in accordance with the declaration of Helsinki. All participants gave written informed consent.

### KL-VS gene analysis

Genotyping was performed at the Institute of Human Genetics, University Hospital of Bonn, Germany using Illumina GWAS microarrays (Omni1-Quad, OmniExpress, OmniExpress v1.0, HumanCoreExomeB). Genotypes of the study participants were imputed with IMPUTE v2.3.1 using the 1000 Genomes Project (release October 2014) as the reference panel. Thereafter, the imputed data were converted to the PLINK ped format using the threshold ≥ 0.8. The Klotho SNPs rs9527025 and rs9536314 were selected from the imputed data in 2559 subjects. For SNP rs9536314 we found 1929 (75.4%) subjects homozygous for the wild type allele, 584 (22.8%) heterozygous and 46 (1.8%) homozygous for the other allele. For SNP rs9527025 we found 1910 individuals homozygous for the wild type allele (74.6%), n = 603 (22.6%) heterozygous and n = 46 (1.8%) homozygous for the other allele. The minor allele frequency (MAF) in our data was 0.132, which is close to 1000 Genomes Project data (https://www.ncbi.nlm.nih.gov/snp/rs9536314) of 0.13 and those of the Mengel-From study with 0.15^14^. We observed discrepant genotypes in 19 individuals, who were heterozygous at rs9527045 and homozygous for the wild type allele at rs9536314. Given that the two variants are in perfect linkage disequilibrium with each other^2^, this can most likely be explained either by genotyping errors or by the inherently not always perfect imputation. The samples showing discrepant results were excluded from the further analysis, leaving 2540 data sets with KL-VS data for further analysis. While the Klotho homozygous group was small, we restricted analysis and reporting to homozygotes of the wild type allele (non-carrier) and KL-VS heterozygous subjects (carrier). Data on the 46 subjects homozygous for the infrequent allele (27 male/19 female, mean 70.2 years (SD = 8.0), range 55-84 years) on performance across the cognition tests are presented in Supplementary Data Table 1.

### Neuropsychological assessment

The neuropsychological assessment in the third examination of the HNR Study comprised a set of paper–pencil and computer aided assessments^17–20^. Of these assessments we analyzed data from the paper–pencil tests. Data from the clock drawing test^21^ and from a computer-based cognitive assessment are not part of the current analysis. The former is a test more specifically used in dementia diagnostics^21^ and the latter was completed by a smaller number of subjects and was recently reported with regard to normative data^17^. Here, we analyzed data of the following cognitive tasks:

*Verbal memory* The verbal memory task comprises a list of eight words to be recalled immediately and following a delay of about 10 min. Items were taken from the "Nürnberger Alters-Inventar" (NAI), a cognitive test suite for elderly subjects^22^. The score represents the number of correctly reproduced words. Larger numbers indicate better performance. *Verbal fluency* Subjects are asked to produce as many names of animals as possible within one minute. The score gives the number of correctly produced words and measures verbal production speed and executive functioning^23,24^. Larger numbers indicate better performance. *Maze test* Subjects mark a route with a pencil through a maze printed on a sheet of paper (also named as “labyrinth test”). This non-verbal task is taken from the German "Nürnberger Alters-Inventar" (NAI)^22^. The score gives the seconds needed to complete the test. After 180 s, task processing is terminated and the assessment is considered as non-assessable. The Maze test measures visual processing, planning and motor speed. Lower numbers indicate better performance. *Stroop task* In the Stroop task subjects have to read a sheet with color words, name colors of lines, and name colors of incongruent colored words. This tests was administered as short version with one card per condition as used in the NAI^25^. The difference in completion time between the last two conditions in seconds gives the additional interference time needed to inhibit the automatic tendency to name the color word instead naming the color it is printed in. Here we analyzed the interference condition, which is suggested to test the inhibition of automated actions as an aspect of executive functioning and referred to as Stroop interference. Lower numbers indicate better results. *Trail Making Test (TMT)* This test comprises two parts^26^. In TMT part A, dots filled with numbers have to be linked with a pencil on a sheet of paper in ascending order. In TMT part B, numbers and letters have to be linked ascending in an alternating order (1, A, 2, B, 3…). The score is the number of seconds needed to complete the task. The limit for valid task completion is 180 s for part A and 300 s for part B. The TMT part A measures visual attention and motor speed, TMT part B measures additional working memory capacity together with executive functions needed to repeatedly switch between numbers and letters^27^. Lower numbers indicate better results in both TMT subtests.

When combining genetic data with those from the neuropsychological tests, we found 1812 subjects with valid haplotypes as well as at least one neuropsychological test result (mean age 68.9 (7.4); 899 male, 913 female). Additional invalid test data due to exceeded time limits in at least one test were found in 157 subjects (Maze = 40, TMT part A = 4/part B = 143).

### Statistical analyses

Changes in cognitive performance related to Klotho KL-VS haplotype (homozygous for the wild type allele vs. heterozygous KL-VS) were assessed by cognition test means in the whole sample, and in groups stratified for age and sex. From age–sex adjusted linear regression models with interaction terms (Klotho-VS-haplotype × sex, and KL-VS-haplotype × age, respectively), we estimated interaction contrasts with 95% CI as measures of effect modification. For example, if differences between KL-VS haplotype carriers and non-carriers are estimated separately for men and for women, the interaction contrast is the difference of these two differences. In the tables, we report means, 95% confidence intervals and Cohen’s d. Cohen’s d was calculated by (mean1[Wild Type]-mean2[Klotho KL-VS])/pooled standard deviation^28^. The differences between KL-VS haplotype and wild type haplotype were transformed into Z-scores, and inverted where needed in order to show adjusted Klotho KL-VS effects. Z-scores were calculated by (individual score − mean score [wild-type subjects]/standard deviation of mean in wild-type subjects). This transformation results in scores demonstrating the deviation of KL-VS subjects from wild-type subjects within a z-distribution which adjusts the wild-type subjects score results to a mean of 0 and a standard deviation of 1. As a result, the KL-VS subjects z-score results are adjusted to a normal distribution and render them comparable across their individual test result ranges^29^. All analyses were done using SPSS (IBM Inc.).

## Results

**Figure 2:**
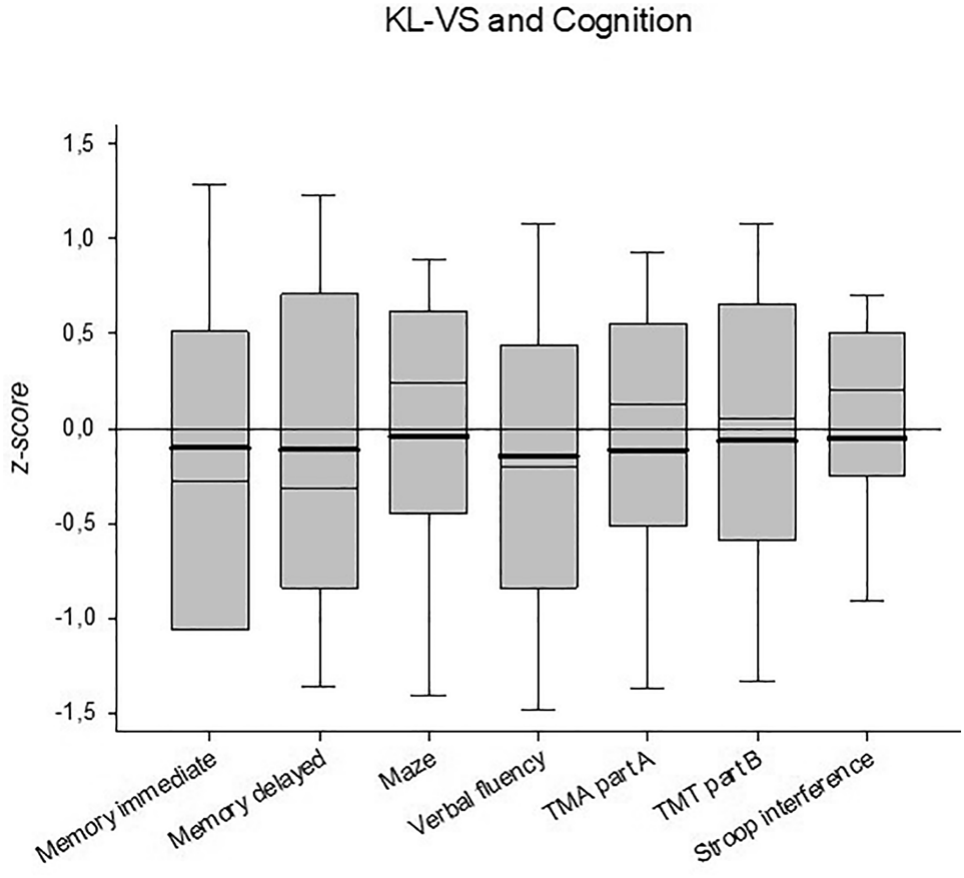
Z-scores for the effect of Klotho KL-VS haplotype in seven tests measuring cognitive abilities. Thin lines represent median data. Thick lines represent mean data. For each test, data of subjects with Klotho wild type haplotype were Z-transformed to a mean of zero and standard deviation of one taken from subjects with KL-VS wild type haplotype. Z-scores give the deviation in subjects with heterozygous Klotho KL-VS haplotype from wild-type subjects. Negative values represent declined performance across all tests.

We compared Klotho KL-VS heterozygous subjects to those homozygous for the wild-type haplotype in the overall analysis. Klotho KL-VS haplotype was consistently associated with lower cognitive performance in all tests (Table 1, Fig. 2). Absolute differences were low, and slightly larger numbers where accompanied by larger CI's, for example in the TMT part B where KL-VS heterozygous subjects needed more time to finish the task (109.5–112.5 s and 95% CI 106.9–112.0 to 107.8–117.1 s). Z-scores (Fig. 2) ranged from Z = − 0.13 to Z = − 0.34. Z-scores for immediate and delayed memory were − 0.024 (SD = 1.0) and − 0.026 (SD = 1.0), maze = − 0.009 (SD = 1.0), verbal fluency − 0.034 (SD = 1.0), TMT part A = − 0.026 (SD = 1.0), TMT part B = − 0.015 (SD = 1.0), stroop interference − 0.013 (SD = 1.0).

**Table 1:** Results in cognitive tests by Klotho haplotype (wild-type vs. KL-VS heterozygous), and difference of test results between KL-VS haplotype and wild type haplotype (95% CI): the Heinz Nixdorf Recall Study.

	KL wild-type		KL-VS heterozygous			Cohen’s d
	Mean (95% CI)	*N*	Mean (95% CI)	*N*	Difference^a^ (95% CI)	
Memory, immediate (n)	5.35 (5.29–5.42)	1392	5.22 (5.10–5.35)	419	− 0.13 (− 0.27; 0.01)	0.10
Memory, delayed (n)	3.63 (3.52–3.73)	1392	3.41 (3.24–3.59)	420	− 0.22 (− 0.42; − 0.01)	0.12
Verbal fluency, animals (n)	23.24 (22.91–23.57)	1393	22.33 (21.73–22.93)	419	− 0.92 (− 1.60; − 0.39)	0.26
Maze (s)	51.27 (49.87–52.67)	1356	52.38 (49.86–54.90)	407	1.11 (− 1.80; 4.01)	− 0.04
TMT A (s)	43.40 (42.41–44.39)	1379	45.53 (43.62–47.44)	417	2.13 (0.04; 4.21)	− 0.11
TMT B (s)	109.46 (106.91–112.01)	1289	112.47 (107.82–117.13)	374	3.01 (− 2.35; 8.36)	− 0.07
Stroop interference (s)	26.91 (25.85–27.97)	1362	28.05 (25.99–30.11)	411	1.14 (− 1.10; 3.38)	− 0.06

^a^Difference values; tests: memory: list of eight words, number of immediate and delayed recalled items, verbal fluency: animal names produced within 1 min, Maze: paper pencil Maze: time to solution, TMT A: Trail Making Test part A: time to solution in seconds, TMT B: Trail Making Test part B: time to solution in seconds, Stroop interference: Stroop test naming the color of different colored color-name minus naming colored bars in seconds. Higher numbers equal better performance in the memory and the verbal fluency task. Lower numbers equal better performance in the Maze, TMT part -A and B and the Stroop interference task.

Compared to men, women showed slightly better results in all tests but the Maze and the TMT part A (Table 2). The difference was small in the TMT part A (43.6 versus 43.2 s) but more substantial with regard to the Maze performance (53.5 versus 49.0 s). In all tests, men and women performed worse when they were carriers of the KL-VS haplotype with the exception of the TMT part B, with slightly shorter completion times in men and longer completion times in women. The largest detrimental effect was seen in females in the verbal fluency task and the TMT part B. Apart from the TMT part B, there were no strong modifications by sex and Klotho gene status.

**Table 2:** Results in cognitive tests by Klotho haplotype (wild-type vs. KL-VS heterozygous) stratified by sex: the Heinz Nixdorf Recall Study.

	Sex	KL wild type				KL-VS heterozygous		
		Mean (95% CI)	*N*	Mean (95% CI)	*N*	Difference^a^ (95% CI)	Cohen’s d	Interaction contrast^a^ (95% CI)
Memory, immediate (n)	Male	5.23 (5.14; 5.32)	682	5.05 (4.88; 5.21)	217	− 0.18 (− 0.38; 0.01)	0.14	0.12 (− 0.16; 0.40)^b^
	Female	5.48 (5.38; 5.57)	710	5.42 (5.23; 5.60)	202	− 0.06 (− 0.26; 0.14)	0.05	
Memory, delayed (n)	Male	3.35 (3.21; 3.49)	682	3.22 (2.99; 3.45)	217	− 0.13 (− 0.41; 0.15)	0.07	− 0.14 (− 0.56; 0.27)
	Female	3.89 (3.75; 4.03)	710	3.62 (3.34; 3.89)	203	− 0.27 (− 0.58; 0.03)	0.14	
Verbal fluency, animals (n)	Male	23.18 (22.69; 23.67)	683	22.65 (21.77; 23.54)	217	− 0.52 (− 1.52; 0.47)	0.08	− 0.81 (− 2.17; 0.56)
	Female	23.30 (22.86; 23.75)	710	21.98 (21.17; 22.78)	202	− 1.33 (− 2.27; − 0.39)	0.20	
Maze (s)	Male	49.03 (47.27; 50.80)	672	50.07 (46.88; 53.27)	214	1.04 (− 2.57; 4.65)	− 0.04	0.42 (− 5.37; 6,21)
	Female	53.47 (51.32; 55.63)	684	54.94 (50.98; 58.89)	193	1.46 (− 3.10; 6.03)	− 0.05	
TMT A (s)	Male	43.22 (41.93; 44.51)	679	44.34 (41.76; 46.93)	215	1.13 (− 1.59; 3.84)	− 0.06	2.08 (− 2.08; 6.25)
	Female	43.58 (42.08; 45.08)	700	46.79 (43.96; 49.62)	202	3.21 (0.04; 6.38)	− 0.16	
TMT B (s)	Male	111.84 (108.02; 115.66)	638	110.71 (104.22; 117.21)	195	− 1.13 (− 8.91; 6.65)	0.02	8.38 (− 2.32; 19.09)
	Female	107.14 (103.75; 110.53)	651	114.39 (107.67; 121.11)	179	7.25 (− 0.10; 14.60)	− 0.16	
Stroop interference (s)	Male	28.03 (26.39; 29.68)	672	28.99 (26.13; 31.85)	210	0.96 (− 2.39; 4.30)	− 0.05	0.27 (− 4.20; 4.74)
	Female	25.84 (24.49; 27.18)	697	27.06 (24.08; 30.05)	201	1.23 (− 1.74; 4.20)	− 0.07	

^a^Interaction contrast values calculated for the interaction between sex and KL-VS haplotype, tests: memory: list of 8 words, number of immediate and delayed recalled items, verbal fluency: animal names produced within 1 min, Maze: paper pencil, time to solution in seconds, TMT A: Trail Making Test part A: time to solution in seconds, TMT B: Trail Making Test part B: time to solution in seconds, Stroop interference: Stroop test naming the color of different colored color-name minus naming colored bars in seconds.

^b^For immediate memory, an interaction contrast of 0.12 means that the difference between wild type and KL-Vs heterozygous in women is 0.12 recalled words higher than the corresponding difference in men.

Analysis of age revealed consistently and substantially lower cognition scores with increasing age across all tasks in both groups of the KL-VS haplotype, up to roughly 40%, e.g. in the delayed memory task and the Stroop interference time (Table 3). When directly comparing the wild type haplotype with KL-VS heterozygous subjects, in immediate and delayed memory, the Maze and the Stroop interference time, the 55–65 years old KL-VS group demonstrated virtually unchanged performance. Memory for example changed by only 0.07 and 0.05 words in the immediate and the delayed word reproduction. Slight changes were also seen in the oldest group ≥ 76 years in the Stroop interference task (0.73 s improvement) and the TMT part A (1.47 s improvement), with large 95% CIs indicating no substantial change. Sixty-six to 75 years old subjects showed, however, lower performance scores, which were substantial in the immediate (5.3–5.0 words) and delayed memory tasks (3.6–3.1 words), in the verbal fluency task (23.1–21.9 words) and the TMT part A (43.4–48.4 s). The other tests revealed the same trend, but to a lesser extent. Interaction contrasts confirmed these results with substantial interaction differences between the middle (66–75 years) and the younger (55–65 years) group with regard to immediate and delayed memory and the TMT part A.

**Table 3:** Results in cognitive tests by KL-VS haplotype (wild-type vs. KL-VS heterozygous) stratified by age group: the Heinz Nixdorf Recall Study.

	Age (years)	KL wild type		KL-VS heterozygous			Cohen’s d	Interaction contrast^a^ (95% CI)
		Mean (95% CI)	*N*	Mean (95% CI)	*N*	Difference^a^ (95% CI)		
Memory, immediate (n)	**≤ **65	5.79 (5.69; 5.88)	523	5.86 (5.69; 6.03)	149	0.07 (− 0.13; 0.27)	− 0.06	–
	66–75	5.32 (5.22; 5.42)	568	5.03 (4.84; 5.22)	183	− 0.29 (− 0.49; − 0.08)	0.24	Mid − 0.36 (− 0.66; − 0.06)^b^
	**≥ **76	4.68 (4.52; 4.83)	301	4.54 (4.29; 4.79)	87	− 0.14 (− 0.46; 0.18)	0.11	High − 0.21 (− 0.57; 0.15)
Memory, delayed (n)	**≤ **65	4.24 (4.09; 4.40)	523	4.30 (4.03; 4.56)	149	0.05 (− 0.27; 0.37)	− 0.03	–
	66–75	3.63 (3.47; 3.78)	568	3.13 (2.86; 3.39)	184	− 0.50 (− 0.81; − 0.19)	0.27	Mid − 0.55 (− 0.99; − 0.11)
	**≥ **76	2.55 (2.35; 2.76)	301	2.51 (2.15; 2.86)	87	− 0.05 (− 0.47; 0.38)	0.02	High − 0.10 (− 0.64; 0.44)
Verbal fluency, animals (n)	**≤ **65	24.96 (24.43; 25.49)	523	24.64 (23.72; 25.56)	149	− 0.32 (− 1.44; 0.79)	0.05	–
	66–75	23.14 (22.64; 23.63)	568	21.91 (21.00; 22.82)	184	− 1.23 (− 2.24; − 0.22)	0.20	Mid − 0.91 (− 2.39; 0.58)
	**≥ **76	20.47 (19.81; 21.13)	302	19.22 (18.00; 20.45)	86	− 1.25 (− 2.64; 0.15)	0.22	High − 0.92 (− 2.74; 0.89)
Maze (s)	**≤ **65	44.01 (42.03; 45.99)	519	42.97 (39.49; 46.45)	146	− 1.04 (− 5.20; 3.11)	0.05	–
	66–75	51.86 (49.84; 53.87)	556	54.74 (51.00; 58.48)	180	2.88 (− 1.24; 7.00)	− 0.12	Mid 3.93 (− 2.34; 10.19)
	**≥ **76	63.54 (59.91; 67.18)	281	64.11 (57.82; 70.40)	81	0.57 (− 6.97; 8.11)	− 0.02	High 1.62 (− 6.14; 9.36)
TMT part A (s)	**≤ **65	36.48 (35.37; 37.59)	521	37.05 (34.85; 39.24)	149	0.57 (− 1.81; 2.95)	− 0.04	–
	66–75	43.43 (42.00; 44.85)	565	48.44 (45.45; 51.43)	184	5.01 (2.00; 8.03)	− 0.28	Mid 4.45 (0.08; 8.81)
	**≥ **76	55.68 (52.98; 58.38)	293	54.20 (49.42; 58.98)	84	− 1.47 (− 7.11; 4.17)	0.06	High − 2.04 (− 7.41; 3.33)
TMT part B (s)	**≤ **65	91.72 (88.61; 94.84)	505	91.79 (85.53; 98.04)	141	0.06 (− 6.68; 6.81)	0.00	–
	66–75	113.71 (109.89; 117.54)	536	118.34 (111.30; 125.39)	136	4.63 (− 3.30; 12.56)	− 0.10	Mid 4.57 (− 6.55; 15.68)
	**≥ **76	136.40 (129.59; 143.22)	248	140.47 (130.27; 150.68)	70	4.07 (− 9.82; 17.96)	− 0.08	High 4.00 (− 10.08; 18.09)
Stroop interference (s)	**≤ **65	20.75 (19.82; 21.67)	520	20.48 (19.10; 21.87)	149	− 0.26 (− 2.14; 1.61)	0.03	–
	66–75	26.79 (25.40; 28.18)	556	30.01 (26.66; 33.36)	179	3.22 (0.13; 6.31)	− 0.18	Mid 3.49 (− 1.30; 8.26)
	**≥ **76	38.13 (34.59; 41.67)	292	37.40 (31.21; 43.58)	83	− 0.73 (− 8.13; 6.66)	0.03	High − 0.47 (− 6.33; 5,40)

^a^Interaction contrast. and interaction difference values, contrasts 55–65 years vs. 66–75 years and 55–65 years vs. 76–87 years; tests: memory: list of 8 words, number of immediate and delayed recalled items, verbal fluency: animal names produced within 1 min, Maze: paper pencil Maze: time to solution, TMT A: Trail Making Test part A: time to solution in seconds, TMT B: Trail Making Test part B: time to solution in seconds, Stroop interference: Stroop test naming the color of different colored color-name minus naming colored bars in seconds.

^b^For immediate memory, an interaction contrast of − 0.36 means that the difference between wild type and KL-Vs heterozygous in age group 66–75 years is 0.36 recalled words lower than the corresponding difference in age group ≤ 65 years.

## Discussion

Analysis of KL-VS haplotype effects on cognition in participants of the Heinz Nixdorf Recall Study revealed partly unexpected results. In the whole sample of 1812 participants, the heterozygous KL-VS haplotype had a consistent but only slightly negative effect on cognition in all cognitive areas under study. This comprised verbal memory, visual attention, motor speed and executive functions including word production, inhibitory control and working memory. Cohen’s d results showed that there was a small effect size related to better word production among Klotho wild-type subjects. This is in contrast to our expectations which we based on the results in the Dubal^8^ study. In the detailed supplementary data to this study, authors reported in the largest cohort nearly unchanged cognition scores with verbal and visual memory, but heterozygous KL-VS dependent improvements in working memory, verbal fluency tasks and a modified Trail Making Test score, indicating that executive function may profit more with KS-VS haplotype as compared to standard memory function. This, however, could not be replicated in our study. The large Danish cohort study in very old subjects in their 9th decade by Mengel-From et al.^14^ reported reduced cognition screening scores from the Mini-Mental-State Examination^30^ when data were adjusted for sex and age. The assessment of an additional composite cognition score yielded no KL-VS related change. This composite score comprised memory and executive functions which included immediate and delayed word list memory, verbal fluency and digit span forward and backward. Scores were lower in KL-VS haplotype subjects, but the result did not reach statistical significance and overall this study did not point toward a cognition improving effect of Klotho genotype among very old subjects. In the Morar et al.^13^ study, authors assessed data from an elderly (mean 76.6 years) population-based cohort of men and reported an indication of cognition improvement in this sample. Taken together, while our study increases the previously reported sample size by more than 26%, evidence accumulates for a lack of a cognition improvement among KL-VS heterozygous older subjects.

### Effect modification by sex

Women showed slightly better results in most tests. Men were just slightly faster in the Maze task and the TMT part A, both comprising motor speed and visual scanning. These results are close to sex differences in cognition in general^27,31^. Sex specific KL-VS haplotype and Cohen’s d results showed that there is little effect modification of the association between KL-VS haplotype and cognition by sex. This is in concordance with Dubal et al.^8^ who reported that gender*KL-VS interaction terms were not significant in their linear regression models. The Morar et al. study assessed only men in their elderly healthy control sample^13^ and the Mengel-From study^14^ did not explicitly assess sex effects. When assessing circulating Klotho in the blood, Crasto et al.^32^ reported no sex specific effects on daily activities. Overall, our data and those by others do not indicate a major Klotho effect modification by sex.

### Effect modification by age

Analysis of age groups revealed an age dependent decline in cognitive function in both haplotypes. Overall, this decline is expected and may start in young adulthood with slower speed performance, followed by accelerating declines in memory and reasoning, whereas vocabulary knowledge peaks in the decade of the 1960s^33^. When analyzing Klotho KL-VS specific effects across age groups, we found performance decreases mostly in the middle age group of 66–75 years old subjects with regard to memory, verbal fluency and the TMT part A with Cohen’s d in the range of |d| ≥ 0.20 indicating a small effect^28^. Accordingly, for memory and TMT part A, interaction contrasts were strongest for the comparison of the 66–75 years with the ≤ 65 years age group. In all tasks but the verbal fluency task, the negative effect of the heterozygous KL-VS haplotype on cognitive performance was smaller in the oldest group compared to the middle age group, indicating a non-linear response of KL-VS haplotype on cognition across age. The Dubal^8^ study did not find a significant Klotho KL-VS × Age interaction in their regression analysis. A more interesting result emerged among younger adults with the Morar^13^ study. This study was designed to assess Klotho KL-VS gene effects among schizophrenia patients. Interestingly, authors reported positive effects of heterozygous Klotho haplotype on memory but not on a general cognition score in middle aged (40.3 years) healthy control subjects. The positive effect of Klotho KL-VS on memory disappeared in a second, older (76.7 years) healthy control group in this study. Taken together, these results indicate that positive effects of heterozygous Klotho KL-VS can be present among younger adults but may disappear with higher age. However, recent data on 1387 children and adolescents between age 3 and 21 years do not support this idea. De Vries et al.^34^ only found a cognition enhancing effect of KL-VS carriers among children up to the age of about 11 years, a result which was not replicated by an independent second sample of 2306 6–12-year-old children. One idea coming from these partly inconsistent results is that more data from younger or middle-aged adults are needed before a final conclusion may be drawn. Another hypothesis is that in humans, KL-VS haplotype may not be a sufficient modificator of cognition, and epigenetic or other biological interactions may have to be considered in order to more sufficiently understand Klotho effects on cognition in humans. Indeed, recent studies assessed this kind of interactions with regard to Amyloid-beta deposition, APOE gene and Alzheimer’s disease^35–37^ and with regard to the idea that Klotho methylation in blood modulates stress responses and thereby the expression of symptoms of post-traumatic stress disorder^38^. The assessment of blood Klotho may be another path to understand the effects of Klotho on cognition in humans^10,11,32^.

### Limitations and strengths

Some limitations have to be considered regarding our study. First, cognitive data originate from the third examination of the Heinz Nixdorf Recall Study. During the course of the study, we might have lost older, more frail and morbid individuals resulting in lager CIs for the older age group and some missing data due to the amount of assessments and their difficulty. Second, due to the cross-sectional analyses, it is not possible to predict if participants with the heterozygous KL-VS haplotype might show a steeper cognitive decline in the further course of the study. As our study cohort comprises participants from 55 to 87 years of age, additional data on younger subjects as in the Morar et al.^13^ study may be helpful to draw further conclusions on the association of the heterozygous KL-VS haplotype among the entire life span. While we based our statistics on changes in mean values in relation to confidence intervals, we did not correct these for multiple comparisons as used in traditional p-value statistics. The problem of alpha error accumulation does not come up in our study. Overall, our data are based on a large sample of subjects from a community-based study. Results however, still have to be interpreted with some caution.

However, our study does have several strengths. Participants were randomly selected from mandatory registries, which reduced the selection or volunteer bias. As discussed above, the fact that subjects in our study have been analyzed with no exclusions due to medical conditions may be additionally seen as a strength of a population-based study. It brings our results more in line with the Danish study by Mengel-From et al.^14^. Another strength of our study is the analysis of subtests assessing various aspects of cognition. While immediate and delayed memory tasks assess different aspects of the same cognitive system and executive functions (as assessed i.e. in the verbal fluency task) are not fully independent from memory function, neuropsychological test scores tend to correlate, but nonetheless show relative sensitivity to specific cognitive domains^39^. Some of the tests analyzed here as separate subtests have also been used in the Dubal et al. study^8^, but were combined into a single cognition index score. Our study points to the relevance of specific cognitive dimensions i.e. memory and visual attention as differentially related to Klotho KL-VS haplotype across age groups.

## Conclusion

In summary, our study found overall slightly lower cognitive performance in heterozygous KL-VS haplotype subjects in a population-based study comprising elderly subjects aged between 55 and 87 years. The effect of sex was low, and age showed a non-linear effect among elderly subjects in their late 1960s. More data from younger or middle-aged adults are needed to draw further conclusions on the effects of heterozygous Klotho KL-VS haplotype on cognition. The assessment of circulating Klotho in blood and its relation of KL-VS gene status may help to improve our knowledge on how Klotho affects cognition in humans.

## Supplementary Information

Supplementary Table 1.

## Data Availability

The data analyzed in this study is subject to the following licenses/restrictions: the corresponding author has full access to all data in the study and final responsibility for the submission of the article for publication. Due to data security reasons (i.e., data contain potentially participant identifying information), the HNR Study does not allow sharing data as a public use file. Requests to access these datasets should be directed to The Heinz Nixdorf Recall Study at the University of Duisburg-Essen: recall@uk-essen.de.
