# Supplementary material for: Klotho KL-VS haplotype does not improve cognition in a population-based sample of adults age 55–87 years
Source: Sci Rep. 2021 Jul 5;11:13852. doi: 10.1038/s41598-021-93211-x (PMC8257625; doi:10.1038/s41598-021-93211-x)
Supplement: Supplementary file 1 — Supplementary Table 1. [file 41598_2021_93211_MOESM1_ESM.docx]

Supplementary material to manuscript

***Klotho KL-VS haplotype does not improve cognition in a population-based sample of adults age 55 to 87 years***

Bernhard W. Müller^1,2,*^, Anke Hinney^3^, Norbert Scherbaum^1,4^, Christian Weimar^5,6^, Christoph Kleinschnitz^7^, Triinu Peters^3^, Lara Hochfeld^8^, Sonali Pechlivanis^6,9^, Andreas Stang^6^, Martha Jokisch^7^, Bernd Kowall^6^

^1^ Department for Psychiatry and Psychotherapy, LVR-Hospital, University of Duisburg-Essen, University Hospital Essen, Essen, Germany

^2^ Department of Psychology, University of Wuppertal, Wuppertal, Germany

^3^Department of Child and Adolescent Psychiatry, Psychosomatics and Psychotherapy, University of Duisburg-Essen, University Hospital Essen, Essen, Germany

^4^ Department of Addictive Behavior and Addiction Medicine, LVR-Hospital, University of Duisburg-Essen, University Hospital Essen, Essen, Germany

^5^ BDH-Klinik Elzach gGmbH, Elzach, Germany

^6^ Institute for Medical Informatics, Biometry and Epidemiology, University Hospital of Essen, Essen, Germany;

^7^ Department of Neurology, University Hospital Essen, University of Duisburg-Essen, Essen, Germany

^8^ Institute of Human Genetics, University Hospital of Bonn, Bonn, Germany

^9^ Institute for Asthma and Allergy Prevention, Helmholtz Zentrum München, German Research Centre for Environmental Health, Munich, Germany

^*^ Correspondence: Bernhard W. Müller, Tel.: +49 201 7227 0, bernhard.mueller@uni-due.de

**Supplementary material table 1:**

Results in cognitive tests by KL-VS haplotype (KL wild-type vs. KL-VS homozygous subjects):
The Heinz Nixdorf Recall Study.

| Cognition tests * | **KL Wild type** | |  | **KL-VS homozygous** | |
| --- | --- | --- | --- | --- | --- |
|  | **mean (95%- CI)** | ***N*** |  | **mean (95%- CI)** | ***N*** |
| Memory, immediate (n) | 5.35 (5.29 - 5.42) | 1392 |  | 5.28 (4.87-5.70) | 46 |
| Memory, delayed (n) | 3.63 (3.52 - 3.73) | 1392 |  | 3.41 (2.78-4.04) | 46 |
| Verbal fluency, animals (n) | 23.24 (22.91 - 23.57) | 1393 |  | 24.26 (21.69-26.82) | 46 |
| Maze (sec.) | 51.27 (49.87 - 52.67) | 1356 |  | 56.28 (43.61-70.03) | 46 |
| TMT A (sec.) | 43.40 (42.41 - 44.39) | 1379 |  | 43.21 (37.77-48.64) | 46 |
| TMT B (sec.) | 109.46 (106.91 - 112.01) | 1289 |  | 130.97 (107.34-154.61) | 46 |
| Stroop interference (sec.) | 26.91 (25.85 - 27.97) | 1362 |  | 25.92 (20.58-31.26) | 46 |

* cognition tests: memory: list of 8 words, number of immediate and delayed recalled items, verbal fluency: animal names produced within 1 minute, Maze: paper pencil Maze: time to solution, TMT A: Trail Making Test part A: time to solution in seconds, TMT B: Trail Making Test part B: time to solution in seconds, Stroop interference: Stroop test naming the color of different colored color-name minus naming colored bars in seconds.
